# Factors associated with postpartum smoking relapse at early postpartum period of Japanese women in the Japan Environmental and Children’s Study

**DOI:** 10.1265/ehpm.23-00059

**Published:** 2023-09-28

**Authors:** Akane Anai, Kaname Asato, Nozomi Tatsuta, Kasumi Sakurai, Chiharu Ota, Shinichi Kuriyama, Junichi Sugawara, Takahiro Arima, Nobuo Yaegashi, Kunihiko Nakai, The Japan Environment and Children’s Study Group

**Affiliations:** 1 Department of Development and Environmental Medicine, Tohoku University Graduate School of Medicine, 2-1 Seiryo-machi, Aoba-ku, Sendai, Miyagi 980-8575, Japan; 2 Environmental and Genome Research Center, Tohoku University Graduate School of Medicine, 2-1 Seiryo-machi, Aoba-ku, Sendai, Miyagi 980-8575, Japan; 3 Institute for Asian and Oceanian Studies, Kyushu University, 744 Motooka, Nishi-ku, Fukuoka, Fukuoka 819-0395, Japan; 4 School of Sport and Health Science, Tokai Gakuen University, 21-233 Nishinohora, Ukigai-cho, Miyoshi, Aichi 470-0207, Japan

**Keywords:** Postpartum smoking relapse, Maternal smoking, Smoking cessation during pregnancy, Factor of smoking relapse

## Abstract

**Background:**

Postpartum smoking relapse is a serious public health concern. Previous studies have identified several risk factors for postpartum smoking relapse; however, very little is known about the predictors of early postpartum smoking relapse. This study aimed to determine postpartum smoking relapse status and its associated risk factors at 1 month postpartum among Japanese women.

**Methods:**

Data were obtained from 93,851 mothers with live births in an ongoing birth cohort study, the Japan Environment and Children’s Study. Data on smoking status and confounding variables were collected using self-administered questionnaires and medical record transcripts. Self-administered questionnaires were administered during the first trimester, second/third trimester, and 1 month after delivery. A multiple logistic regression analysis was performed.

**Results:**

Among the 14,326 mothers who smoked during pregnancy, 10,917 (76.2%) quit smoking during pregnancy. Subsequently, 617 (5.7%) of the mothers who had quit relapsed smoking at 1 month postpartum. Maternal age (≤24, ≥35), maternal education (≤12 years), parity (≥Second), feeding method (Formula milk), partner smoking status during pregnancy (Smoker), number of cigarettes per day before the cessation of smoking (≥11), maternal alcohol consumption at 1-month postpartum (Drinker), postpartum depression (EPDS score ≥9), and spending time at the parents’ home after delivery (≥14 days) were associated with smoking relapse.

**Conclusions:**

A certain number of mothers relapsed even 1 month postpartum. Besides mother's alcohol and smoking habit before pregnancy, breastfeeding and partner smoking are important factors in early postpartum smoking relapse in Japan.

## Introduction

Maternal smoking during pregnancy is a critical public health issue. Active smoking increases the risk of some cancers [1–3] and other preventable diseases such as cardiovascular diseases, respiratory diseases, and diabetes mellitus [4, 5]. Smoking during pregnancy increases the risk of hypertensive disorders of pregnancy [6] and postpartum depression [7]. In addition, it is well known that maternal smoking during pregnancy increases not only the risk of adverse birth outcomes, such as low birth weight [8, 9], stillbirth, neonatal death, perinatal death [10], and preterm birth [11], but also their health risks such as atopic eczema [12], wheezing, and asthma [13]. Under these circumstances, pregnancy is an opportunity for women to quit smoking. Previous studies reported that 33.9–53.0% of women who smoked quit smoking when they become pregnant [14–16] and it is 66.5–77.7% in Japan [13, 17, 18].

However, some mothers who quit then resume smoking after giving birth, and this relapse causes postnatal exposure to tobacco smoke among infants. Postnatal secondhand exposure to tobacco smoke increases the risk for various childhood illnesses, including asthma [19, 20], severe bronchiolitis [21] and attention-deficit/hyperactivity disorder symptoms [22]. It has been reported that 22.5% of women who quit smoking during their pregnancy relapsed at 3–4 months postpartum and 43.4–70.3% of that after 18 months postpartum in Japan [17, 18]. Therefore, efforts to keep mothers who quit smoking abstinent after childbirth are also extremely important.

Several previous studies have described the risk factors for maternal smoking relapse. According to a systematic review conducted by Orton et al. [23], being less educated, younger maternal age, multiparity, living with a partner or household member who smoked, experiencing higher stress, depression or anxiety, not breastfeeding, intending to quit only for pregnancy, and low confidence to remain abstinent postpartum were significant predictors of postpartum smoking relapse. Similarly, a shorter breastfeeding period, younger maternal age, women who were employed at the time of survey, women having a partner who smoked after giving birth were positive predictors for maternal postpartum relapse, and women spending time with their child in a relaxed mood and women having someone to talk to on the Internet about childrearing were negative predictors in the Japanese studies [17, 18].

Furthermore, early postpartum smoking relapse immediately after childbirth is an additional serious issue because of the exposure of infants to tobacco-derived chemical compounds through breastfeeding. Indeed, nicotine, cotinine, and 3-hydroxycotinine have been detected in smoking mothers’ breast milk [24]. Maternal smoking during lactation increases the risk of sudden infant death syndrome, neurodevelopmental and behavioral disorders, and sleep disruption in offspring as short-term health outcomes [25]. Previous studies conducted in the United Kingdom and United States reported that 46.5% of women who quit smoking during pregnancy relapsed smoking at 6 weeks postpartum in the United Kingdom, and 27.2% at 1 month postpartum in the United States [26, 27]. In those studies, women who lived in deprived urban areas, had three or more children, had other smokers in the household, not intending to remain abstinent and lower quitting confidence were included as predictor of early postpartum smoking relapse. In addition, breastfeeding-related variables are also important factors; women who were breastfed were significantly less likely to relapse in the United Kingdom, and women not planning to breastfeed at 1 month postpartum were significantly more likely to relapse in the United States [26, 27].

However, the smoking relapse rate in the early postpartum period and its predictors have not yet been clarified in Japan. Therefore, the first purpose of the present study was to determine the rate of postpartum smoking relapse at 1 month postpartum in Japan using a nationwide large birth cohort study. The second purpose was to clarify the risk factors associated with smoking relapse at 1 month postpartum. This information will be useful to keep mothers abstinent from smoking relapse in the early postpartum period.

## Methods

### Study design and participants

**Fig. 1:**
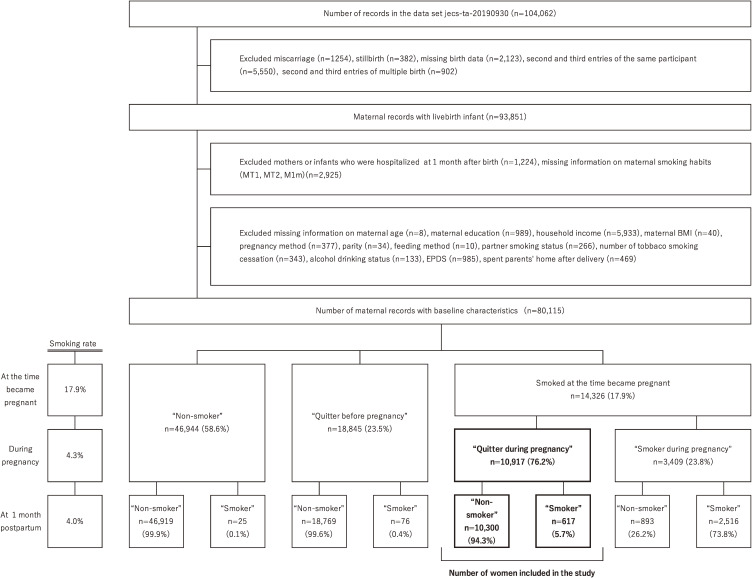
Flow chart for selection of women from JECS and participants’ smoking status. MT1, self-administered questionnaires conducted during the first trimester of pregnancy; MT2, self-administered questionnaires conducted during the second/third trimester of pregnancy; M1m, self-administered questionnaires conducted at one month after delivery; BMI, Body Mass Index; EPDS, Edinburgh Postnatal Depression Scale.

The methodology and protocol of the Japan Environment and Children’s Study (JECS) have been previously described in detail [28, 29]. The JECS is an ongoing nationwide birth cohort study, which is a national project funded directly by the Ministry of Environment to elucidate the influence of environmental factors on health. Between January 2011 and March 2014, we registered >100,000 pregnant women. Written informed consent was obtained from all the participants. The present study was based on the jecs-ta-20190930 dataset, which includes 104,062 fetal records, and was released in October 2019. From this dataset, women with miscarriage, stillbirth, missing birth data, second and third entries of the same participant, second and third entries of multiple births, mothers or infants hospitalized at 1 month after birth, missing information on maternal smoking habit during pregnancy, or self-administered questionnaires 1 month after delivery were excluded. We further excluded missing data on maternal age, maternal education, household income, maternal body mass index (BMI), fertility treatment status, parity, feeding method, partner smoking status during pregnancy, number of cigarettes smoked before quitting smoking, alcohol drinking status 1 month after delivery, score of Edinburgh Postnatal Depression Scale (EPDS) [30] and time spent at parents’ home after delivery. Women who quit smoking during pregnancy were included in this study, and 10,917 women were analyzed to determine the risk factors associated with postpartum smoking relapse 1 month after delivery (Fig. 1). The JECS protocol was reviewed and approved by the Ministry of the Environment’s Institutional Review Board on Epidemiological Studies and the Ethics Committees of all participating institutions (Ethical Number: No. 100910001).

### Data collection and variables

Outcome data and confounding variables were collected from self-administered questionnaires and medical record transcripts. Self-administered questionnaires were administered during the first trimester (MT1), second/third trimester (MT2), and one month after delivery (M1m).

Associated risk factors for postpartum smoking relapse which were reported by prior studies are included as independent variables; maternal age at delivery (<20, 20–24, 25–29, 30–34, 35–39, or ≥40), maternal education (≤12 years, or ≥13 years), annual household income (<2, 2–3.9, 4–5.9, 6–7.9, or ≥8 million JPY), pre-pregnancy BMI (<18.5, 18.5–24.9, or ≥25.0 kg/m^2^), parity (first, or ≥second), feeding method (breastmilk, breastmilk and formula milk, or formula milk), status of multiple birth (single birth or multiple birth), number of cigarettes smoked before cessation (1–10/day, ≥11/day), alcohol drinking status at 1 month after delivery (non-drinker, drinker), partner smoking status during pregnancy (Non-smoker, quitter before pregnancy, quitter during pregnancy, smoker) and maternal depression status after delivery (EPDS score <9, or ≥9). In addition to these factors, the status of spending time at parents’ home after delivery (did not spend at parents’ home or spent <14 days, or spent ≥14 days) was examined as a risk factor for postpartum smoking relapse. Finally, because women who underwent infertility treatment were less likely to smoke than those who did not [31], the status of infertility treatment (natural treatment, or infertility treatment) was included as an independent variable in this study.

Maternal and partner smoking status during pregnancy (MT1 and MT2) were scored on the questionnaire as “Non-smoker,” “Quitter before pregnancy,” “Quitter during pregnancy” or “Smoker.” Data on maternal and partner smoking status during pregnancy were based on the MT2 questionnaire, with results from the MT1 questionnaire used to complement the maternal smoking status when it was missing from the MT2 questionnaire. Maternal smoking status at 1 month after childbirth was scored on the M1m questionnaire as “Non-smoker,” “Quitter before pregnancy,” “Quitter during pregnancy,” “Smoker (1–10 pieces per day),” “Smoker (11–20 pieces per day)” or “Smoker (21 pieces or more per day).” We combined “Non-smoker,” “Quitter before pregnancy” or “Quitter during pregnancy” as the “Non-smoker” after delivery, while others including “Smoker (1–10 cigarettes/day),” “Smoker (11–20 cigarettes/day)” or “Smoker (21 pieces or more per day)” were the “Smoker” at 1 month after delivery. Among “Quitter during pregnancy” women based on MT1 and MT2 questionnaires, those who were the “Non-smoker” at M1m were classified as the postpartum maintained cessation group, and those who were the “Smoker” at M1m classified as the postpartum smoking relapse group (Fig. 1).

Data on maternal education and annual household income were obtained using the MT2 questionnaire. Pre-pregnancy BMI was calculated using the formula: kg/m^2^. Data on parity and feeding methods were based on medical record transcripts, with results from the MT1 questionnaire for parity or the M1m questionnaire for feeding methods to complement when they were missing. Data on the number of cigarettes smoked before cessation were based on the MT2 questionnaire, with results from the MT1 questionnaire used to complement the maternal smoking status. Data on alcohol consumption at 1 month after delivery, status of spent parents’ home after delivery, and maternal depression after delivery were obtained from the M1m questionnaire. The Japanese version of the Edinburgh Postnatal Depression Scale (EPDS) was used to assess maternal postpartum depression [30]. The EPDS is a self-administered 10-item scale used to screen for postpartum depression [32]. In this study, an EPDS score of ≥9 was defined as postpartum depression [30, 33].

### Data analysis

Univariate associations between maternal postpartum smoking status and variables were examined using the Chi-square test, as appropriate. Variables with a Chi-square test of p < 0.05 were selected as covariates for multiple logistic regression analyses to identify predictors of smoking relapse in the early postpartum period. All statistical analyses were performed using JMP^®^ 16 software (SAS Institute Inc., Cary, NC, USA).

## Results

Smoking rates at the time of pregnancy, during pregnancy, and after delivery were 17.9%, 4.3%, and 4.0%, respectively. Among women who smoked at the time of pregnancy, 76.2% quit smoking during pregnancy. Among these women, 94.3% maintained smoking cessation, and the remaining 5.7% relapsed smoking at 1 month postpartum (Fig. 1).

Table 1 shows the characteristics of the 10,917 women who quit smoking during pregnancy. The majority of the women were 25–34 years old (63.3%), and just over half of the women were first-time mothers (51.2%). More than half of the women had ≤12 years of education (56.6%), and the most frequent household income was 2–3 million JPY (44.9%). Thirty-six percent of the women had smoked ≥11 cigarettes/day before smoking cessation. In total, 7.3% of women had been drinking alcohol 1 month after delivery. The majority of women reported that their partner had been smoking during their current pregnancy (74.3%).

**Table 1:** Characteristics of women who quit smoking during pregnancy.

	**Total** **(n = 10,917)**		**Cessation** **(n = 10,300)**		**Relapse** **(n = 617)**		***p***
		
	**n**	**%**	**n**	**%**	**n**	**%**	
Maternal age at delivery (yrs)							
<20	145	1.3	127	1.2	18	2.9	<0.0001
20–24	1726	15.8	1615	15.7	111	18.0	
25–29	3594	32.9	3403	33.0	191	31.0	
30–34	3321	30.4	3168	30.8	153	24.8	
35–39	1834	16.8	1716	16.7	118	19.1	
≥40	297	2.7	271	2.6	26	4.2	
Maternal education (yrs)							
≤12	6183	56.6	5740	55.7	443	71.8	<0.0001
≥13	4734	43.4	4560	44.3	174	28.2	
Household income (million JPY)							
<2	1040	9.5	960	9.3	80	13.0	<0.0001
2–3	4902	44.9	4591	44.6	311	50.4	
4–5	3162	29.0	2999	29.1	163	26.4	
6–7	1136	10.4	1098	10.7	38	6.2	
≥8	677	6.2	652	6.3	25	4.1	
Pre-pregnancy BMI (kg/m^2^)							
<18.5	1865	17.1	1764	17.1	101	16.4	0.015
18.5–24.9	7675	70.3	7260	70.5	415	67.3	
≥25	1377	12.6	1276	12.4	101	16.4	
Fertility treatment							
No	10568	96.8	9962	96.7	606	98.2	0.040
Yes	349	3.2	338	3.3	11	1.8	
Status of multiple birth							
Single birth	10845	99.3	10232	99.3	613	99.4	0.972
Multiple birth	72	0.7	68	0.7	4	0.6	
Parity							
First	5586	51.2	5369	52.1	217	35.2	<0.0001
≥Second	5331	48.8	4931	47.9	400	64.8	
Feeding method at 1 month after delivery							
Breast milk	5150	47.2	4961	48.2	189	30.6	<0.0001
Breast and formula milk	5071	46.5	4783	46.4	288	46.7	
Formula milk	696	6.4	556	5.4	140	22.7	
Partner smoking status during pregnancy							
Non-smoker	1138	10.4	1085	10.5	53	8.6	0.001
Quitter before pregnancy	827	7.6	794	7.7	33	5.4	
Quitter during pregnancy	840	7.7	808	7.8	32	5.2	
Smoker	8112	74.3	7613	73.9	499	80.9	
Number of cigarettes use per day							
1–10	7020	64.3	6716	65.2	304	49.3	<0.0001
≥11	3897	35.7	3584	34.8	313	50.7	
Maternal drinking status at 1 months after delivery							
Non-drinker	10122	92.7	9646	93.7	476	77.1	<0.0001
Drinker	795	7.3	654	6.3	141	22.9	
EPDS score							
<9	8894	81.5	8420	81.7	474	76.8	0.002
≥9	2023	18.5	1880	18.3	143	23.2	
Spent at parents’ home after delivery							
Did not spend or <14 days	6108	56.0	5691	55.3	417	67.6	<0.0001
≥14 days	4809	44.1	4609	44.8	200	32.4	

BMI, Body Mass Index; EPDS, Edinburgh Postnatal Depression Scale.

Table 2 shows results from logistic regression analyses examining the association between selected factors and smoking relapse at 1 month postpartum. In the crude model, statistically significant associations were observed for all selected factors; maternal age, maternal education, house hold income, pre-pregnancy BMI, fertility treatment, parity, feeding methods at 1 month after delivery, partner smoking status during pregnancy, number of cigarettes use per day, maternal drinking status at 1 month after delivery, score of EPDS and spent at parents’ home after delivery. In the adjusted model, women who feed formula milk were more likely to relapse than women who feed only breast milk (adjusted OR = 1.44, 95% CI: 1.19–1.75 for breast and formula milk; adjusted OR = 4.05, 95% CI: 3.15–5.22 for only formula milk). Women who have been drinking alcohol at 1 month after delivery were three times more likely to relapse than women who have not started drinking alcohol (adjusted OR = 3.19, 95% CI: 2.56–3.97). For other factors, women who are aged <20 years were twice as likely to relapse compared to those aged 30–34 years (adjusted OR = 2.77, 95% CI: 1.56–4.93). Significant increase of relapse risk was also observed aged 20–24, 30–34 and ≥40 years (adjusted OR = 1.45, 95% CI: 1.10–1.90 for 20–24 years; adjusted OR = 1.30, 95% CI: 1.01–1.68 for 35–39 years; adjusted OR = 1.68, 95% CI: 1.06–2.65 for ≥40 years). Women who had ≤12 years of education were more likely to relapse than those who had ≥13 years of education (adjusted OR = 1.27, 95% CI: 1.05–1.55). Women who had ≥second parity were more likely relapse than <first parity (adjusted OR = 1.84, 95% CI: 1.51–2.23). There were also significant positive associations between partner smoking status during pregnancy, the number of cigarettes smoked per day, and EPDS scores. Women having partner who had smoked during the current pregnancy were more likely to relapse than who have non-smoker partner (adjusted OR = 1.42, 95% CI: 1.05–1.92). Women who had smoked ≥11 cigarettes per day before their smoking cessation were more likely to relapse than women who had smoked <11 cigarettes per day (adjusted OR = 1.78, 95% CI: 1.50–2.11). Women who had an EPDS score ≥9 were more likely to relapse than those with a score <9 (adjusted OR = 1.28, 95% CI: 1.04–1.57). Spending ≥14 days at parents’ home after delivery was found to be a protective factor for postpartum smoking relapse (adjusted OR = 0.79, 95% CI: 0.66–0.96). Significant associations were not observed with household income, pre-pregnancy BMI and fertility treatment status at the current pregnancy in the adjusted model.

**Table 2:** Crude and adjusted odds ratios for factors associated with postpartum smoking relapse.

	**Relapsed**	**Crude**		**Adjusted**	
		
	**%**	**OR**	**95%CI**	**OR**	**95%CI**
Maternal age at delivery (yrs)					
<20	12.4	2.93	1.75–4.93	2.77	1.56–4.93
20–24	6.4	1.42	1.11–1.83	1.45	1.10–1.90
25–29	5.3	1.16	0.93–1.45	1.22	0.97–1.54
30–34	4.6	reference		reference	
35–39	6.4	1.42	1.11–1.82	1.30	1.01–1.68
≥40	8.8	1.99	1.29–3.07	1.68	1.06–2.65
Maternal education (yrs)					
≤12 years	7.2	2.02	1.69–2.42	1.27	1.05–1.55
≥13 years	3.7	reference		reference	
Household income (million JPY)					
<2	7.7	1.53	1.16–2.02	1.22	0.91–1.64
2–3	6.3	1.25	1.03–1.51	1.14	0.93–1.40
4–5	5.2	reference		reference	
6–7	3.3	0.64	0.44–0.91	0.75	0.52–1.08
≥8	3.7	0.71	0.46–1.08	0.78	0.50–1.22
Pre-pregnancy BMI (kg/m^2^)					
<18.5	5.4	1.00	0.80–1.25	0.97	0.77–1.23
18.5–24.9	5.4	reference		reference	
≥25	7.3	1.38	1.11–1.73	1.17	0.92–1.48
Fertility treatment					
No	5.7	reference		reference	
Yes	3.2	0.53	0.29–0.98	0.75	0.40–1.41
Parity					
First	3.9	reference		reference	
≥Second	7.5	2.01	1.69–2.38	1.84	1.51–2.23
Feeding method at 1 month after delivery					
Breast milk	3.7	reference		reference	
Breast and formula milk	5.7	1.58	1.31–1.91	1.44	1.19–1.75
Formula milk	20.1	6.61	5.22–8.36	4.05	3.15–5.22
Partner smoking status during pregnancy					
Non-smoker	4.7	reference		reference	
Quitter before pregnancy	4.0	0.85	0.55–1.33	1.01	0.64–1.60
Quitter during pregnancy	3.8	0.81	0.52–1.27	0.93	0.59–1.48
Smoker	6.2	1.49	1.22–1.83	1.42	1.05–1.92
Number of cigarettes use per day					
1–10	4.3	reference		reference	
≥11	8.0	1.93	1.64–2.27	1.78	1.50–2.11
Maternal drinking status at 1 months after delivery					
Non-drinker	4.7	reference		reference	
Drinker	17.7	4.37	3.56–5.36	3.19	2.56–3.97
EPDS score					
<9	5.3	reference		reference	
≥9	7.1	1.35	1.11–1.64	1.28	1.04–1.57
Spent at parents’ home after delivery					
Did not spend or <14 days	6.8	reference		reference	
≥14 days	4.2	0.59	0.50–0.70	0.79	0.66–0.96

OR, Odds ratio; 95% CI, 95% Confidence Interval; BMI, Body Mass Index; EPDS, Edinburgh Postnatal Depression Scale.

## Discussion

This study examined the smoking relapse rate and its associated risk factors during the early postpartum period using Japanese nationwide data. In this study, 5.7% of women who quit smoking during pregnancy relapsed at 1 month postpartum. Women who were feeding formula milk, drinking alcohol at 1 month postpartum, aged ≤24 or ≥35, had ≤12 years of education, had ≥second parity, had a partner who had smoked during the current pregnancy, had smoked ≥11 cigarettes per day before their smoking cessation, and had an EPDS score ≥9 were more likely to relapse smoking. Women spending ≥14 days at their parents’ home after delivery were less likely to relapse smoking in the early postpartum period.

In this study, 14,326 (17.9%) women smoked at the time they became pregnant, and 76.2% quit smoking during pregnancy (Fig. 1). Although this number is slightly higher than that reported in previous studies in Japan (76.2% in this study, 66.5–68.9 in previous studies) [17, 18], our study results support the view of previous studies that pregnancy highly motivated mothers to quit smoking [17, 18]. The smoking relapse rate at 1 month postpartum in this study was 5.6%, which is lower than that reported in previous studies in the US (27.2%) and the UK (46.5%) [26, 27]. Although this number of relapsed women was smaller than that in other countries, it was confirmed that a certain number of women relapsed in the early postpartum period in Japan.

Previous studies have suggested that breastfeeding protects against smoking relapse [27, 34]. Maternal smoking also increases the risk of early weaning [35]. Our findings are consistent with those of previous studies; the relapse rate of women who exclusively breastfed and partially breastfed was lower than that of women who did not breastfeed (Table 1). As a reason for not resuming smoking while breastfeeding, smoking mothers dislike the transfer of tobacco-derived chemicals to their infants. Nicotine, cotinine, and 3-hydroxycotinine have been detected in smoking mothers’ breastmilk [24], and nicotine and cotinine were detected in infant serum and urine from mothers who smoked through breastfeeding [36]. These circumstances are considered to be important obstacles to maternal relapse. The reason of lower smoking relapse rate in this study compere to US and UK may be related to breastfeeding status of subjects. Compared to previous studies, the proportion of breastfeeding among the subjects of this study was overwhelmingly high; 93.6% in this study, 50% in the study of US, 29.4% in the study of UK [26, 27]. Based on these findings, promotion of breastfeeding during maternity courses or prenatal checkups by local governments or clinics may be effective in preventing postpartum smoking relapse. An interventional study conducted by Logan et al. (2017) indicated that promoting breastfeeding is effective in maintaining smoking cessation prior to weaning [37]. Needless to say, exclusive breastfeeding of infants for the first 6 months of their life is recommended by the World Health Organization [38], since breastfeeding has many benefits.

Partner smoking is a predictor of postpartum smoking relapse [16, 17]. A similar result was observed in this study; women with a partner who smoked during pregnancy had a higher risk of postpartum smoking relapse than women with a partner who did not smoke. Our study additionally shows that having partners who quit smoking before or during pregnancy did not increase the risk of postpartum smoking relapse (Table 2). Therefore, the absence of smoking by partners during pregnancy is an important factor for preventing postpartum smoking relapse. Unfortunately, the number of partners who quit smoking during pregnancy is small, with only 9.4% of partners quitting smoking during pregnancy. Further efforts are required to help partners quit smoking before or during pregnancy. Considering that paternal smoking is also related to neonatal secondhand smoke, intervention for a partner smoking is an important issue to prevent maternal postpartum smoking relapse.

The strength of this study lies in its large sample size compared to previous studies. The limitations of this study include the fact that data on smoking status were obtained from self-administered questionnaire surveys instead of biological monitoring. In addition, because this was an observational study, causal relationships could not be concluded. This study indicated that not/less breastfeeding is a predictor of postpartum smoking relapse. However, it is unknown whether breastfeeding prevents maternal smoking relapse or if they do not breastfeed because they want to smoke.

## Conclusions

This is the first study that examined the smoking relapse status during the early postpartum period in Japan using nationwide data. Among the women who smoked at the time they became pregnant, 76.2% quit smoking during pregnancy, and among these women, 5.6% had relapsed smoking at 1 month postpartum. Maternal age (≤24, ≥35), maternal education (≤12 years), parity (≥Second), feeding method (Formula milk), partner smoking status during pregnancy (Smoker), number of cigarettes per day before the cessation of smoking (≥11), maternal alcohol consumption at 1-month postpartum (Drinker), postpartum depression (EPDS score ≥9), and spending time at the parents’ home after delivery (≥14 days) were associated with smoking relapse. Among those predictors associated with maternal postpartum smoking relapse at 1 month postpartum, breastfeeding and partner smoking are important in preventing early postpartum smoking relapse in Japan.

## Abbreviations

JECS: the Japan Environment and Children’s Study
BMI: body mass index
EPDS: Edinburgh Postnatal Depression Scale
MT1: first trimester
MT2: second/third trimester
M1m: one month after delivery

## Declarations

### Availability of data and material

Data are unsuitable for public deposition due to ethical restrictions and legal framework of Japan. It is prohibited by the Act on the Protection of Personal Information (Act No. 57 of 30 May 2003, amendment on 9 September 2015) to publicly deposit the data containing personal information. Ethical Guidelines for Medical and Health Research Involving Human Subjects enforced by the Japan Ministry of Education, Culture, Sports, Science and Technology and the Ministry of Health, Labour and Welfare also restricts the open sharing of the epidemiologic data. All inquiries about access to data should be sent to: jecs-en@nies.go.jp. The person responsible for handling enquiries sent to this e-mail address is Dr Shoji F. Nakayama, JECS Programme Office, National Institute for Environmental Studies.
